# Exposure and Risk Assessment of Second- and Third-Hand Tobacco Smoke Using Urinary Cotinine Levels in South Korea

**DOI:** 10.3390/ijerph19063746

**Published:** 2022-03-21

**Authors:** Jiyeon Yang, Shervin Hashemi, Wonseok Han, Yoojin Song, Youngwook Lim

**Affiliations:** 1 Institute for Environmental Research, Yonsei University College of Medicine, Seoul 03722, Korea; jyyang67@yuhs.ac (J.Y.); shervin@yuhs.ac (S.H.); 2 Graduate School of Public Health, Yonsei University, Seoul 03722, Korea; hnwnsk7@yuhs.ac (W.H.); thddbwls1301@yuhs.ac (Y.S.)

**Keywords:** cotinine, environmental tobacco smoke, excess lifetime cancer risk, hazard index, second-hand smoke, third-hand smoke

## Abstract

Exposure to environmental tobacco smoke (ETS) is the reason for approximately 1% of global mortality. ETS exposure can happen either as inhalation of direct cigarette smoke (second-hand smoke) or its associated residue particles (third-hand smoke), especially when living with a smoker in the same family. This study investigated the association between the urinary cotinine levels, as biomarkers of exposure to tobacco smoke, of smokers and those exposed to second-hand and third-hand smoke while living in the same family, through a Korean nationwide survey. Direct assessment of ETS exposure and its lifetime effect on human health is practically difficult. Therefore, this study evaluated the internal estimated daily intake (I-EDI) of nicotine and equivalent smoked cigarette per day (CPD). The carcinogenic and non-carcinogenic inhalation risks of ETS exposure were assessed by considering the calculated equivalent CPD and composition of cigarette smoke of high-selling cigarette brands in South Korea. The results show that there is a statistically significant positive correlation between the cotinine levels of smokers and those of the non-smokers living in the same family. The risk assessment results yielded that hazard index (HI) and total excess lifetime cancer risk (ECR) for both second-hand and third-hand smoke exposure can exceed 1 and 1 × 10^−6^, respectively, especially in women and children. In the composition of the cigarette smoke, 1,3-butadiene and acrolein substances had the highest contribution to HI and ECR. Consequently, the provision of appropriate plans for smoking cessation as a strategy for the prevention of ETS exposure to women and children is deemed necessary.

## 1. Introduction

Exposure to environmental tobacco smoke (ETS), also known as passive smoking, either as direct exposure to tobacco smoke (second-hand smoke, SHS) or to tobacco-smoke-associated residual particles (third-hand smoke, THS), is estimated to be the cause of approximately 1% of global mortality [1]. According to the World Health Organization (WHO), exposure to passive smoking contributes to around 1.2 million deaths annually [2]. It is estimated that around 50 smokers can contribute to the death of one non-smoker individual who is exposed to SHS [3]. Meanwhile, passive smoking has significant contributions to the incidence of sickle cell disease [4]. In children, passive smoking has hazardous effects on lung and immune functions, and it can increase the severity of cystic fibrosis [5]. Cohort studies show a strong association between SHS and the incidence of lung and breast cancer in women [6].

In order to prevent passive smoking, the WHO has established the Framework Convention on Tobacco Control (FCTC), which emphasizes protection from exposure to tobacco smoke in indoor workplaces, public transport, indoor public places, and, as appropriate, other public places [7,8,9,10]. Meanwhile, strengthening the implementation of the WHO FCTC in all countries is recognized as one of the targets of the United Nations’ third sustainable development goal (SDG 3.a) [11,12].

It is estimated that over 2000 chemicals exist in the composition of tobacco, and among them, at least 250 are known as toxic ones [13,14]. The number of chemicals can be doubled when tobacco is incompletely burned during smoking [13]. Studies show that cigarette smoke comprises significantly higher concentrations of nicotine, tar, carbon monoxide, ammonia, phenol, and nitric oxide than tobacco [14]. Some of these chemicals can contribute to accelerating the metabolism of nicotine. For example, the presence of ammonia can enhance the conversion of nicotine to non-ionized or free-base states [14]. Moreover, chemicals such as 1,3-butadiene, benzene, isoprene, pyridine, and toluene exist in the smoke stream of cigarettes and can cause second-hand or third-hand exposure through inhalation and cause a range of health effects including pulmonary inflammation, incidences of lung cancer, chronic obstructive pulmonary disease (COPD), cardiovascular disease, reproductive and developmental effects, and immune system suppressions [13,14]. In this regard, assessment of exposure to tobacco smoke and its associated risk through considering the chemical composition of tobacco smoke is crucial.

Although indoor smoking is prohibited in the Republic of Korea, exposure to SHS or THS is common because smokers usually smoke outdoors near the buildings, such as restaurants, bars, and department stores, that do not have controlled or well-managed smoking areas [8,9]. Nevertheless, fewer studies are being conducted in South Korea to assess ETS exposure and risk.

There are different methods to assess tobacco smoke exposure for ETS and its associated carcinogen risk [15]. One of these methods is through measuring the tobacco smoke exposure biomarkers, such as cotinine, trans-3’-hydroxycotinine, and 4-(methylnitrosamino)-1-(3-pyridyl)-1-butanol (NNAL), in urine, blood, nails, or hair [1,8]. Among these biomarkers, cotinine is known to be a major metabolite of nicotine through the liver enzyme CYP2A [12,16,17]. Since nicotine is the primary compound in the composition of the ETS, cotinine can be a reliable biomarker for assessing ETS exposure [1,16,18]. Exposure assessment using urinary cotinine is common due to the relatively easier and affordable sampling and analyzing process [17]. As described by Benowitz et al. [19], after considering the metabolism process of nicotine, the daily intake of nicotine for second-hand and third-hand smokers can be estimated with acceptable accuracy. Thereafter, following Marano et al. [20], by considering the composition of the toxic particles in the smoke stream, the carcinogenic and non-carcinogenic risks of exposure to ETS can be assessed.

Accordingly, this study aims to assess (1) the urinary cotinine level of second-hand and third-hand smokers and its correlation with the urinary cotinine level of smokers living in the same family through a nationwide survey, (2) the exposure to ETS by estimating the daily passive intake of nicotine using the level of the biomarker, and (3) the risk of this exposure by considering the chemical composition of the main smoke stream of highly sold cigarettes in South Korea.

## 2. Materials and Methods

### 2.1. Survey of Smoking Status and Tobacco Smoke Exposure Urinary Biomarker

Datasets of the Seventh Korea National Health and Nutrition Examination Survey (KNHANES VII) were used for extracting raw data on the smoking status and tobacco smoke exposure urinary biomarkers on a nationwide scale. Generally, the Korea National Health and Nutrition Examination Survey (KNHANES) is a reliable nationwide survey in Korea, conducted by the Korea Disease Control and Prevention Agency (KDCA). The sampling framework is based on a multi-stage clustered probability design, including geographically defined primary sampling units [21]. For KNHANES, interviews and examinations are conducted face-to-face by trained medical staff and interviewers at the mobile examination centers, and the annual response rate is over 75% [21]. The KNHANES VII was conducted for three years (2016–2018) and the results are open access [22]. This survey included 8150, 8127, and 7992 participants in the years 2016, 2017, and 2018, respectively.

Regarding tobacco smoke exposure, the survey includes two parts aiming at participants who are 6 years or older. The first part is a self-reporting questionnaire with 17 items. First, it identifies smokers (also known as active smokers), former smokers (an adult who has smoked at least 100 cigarettes in his or her lifetime, but who had quit smoking at the time of interview), and non-smokers. Smokers are asked about their smoking habits, such as smoking frequency and the average number of smoked cigarettes per day. Former smokers are asked about their cessation duration and their smoking habits during the time that they used to smoke frequently. In addition, the questionnaire aims to investigate the status of exposure to ETS by asking if the subject is being regularly exposed to second-hand or third-hand smoke in their residential area, working place, or any public places. Finally, the questionnaire targets adolescent participants (aged 12–19) and asks if they have any experience of direct smoking of cigarettes.

The second part of the survey includes measurement of urinary cotinine, NNAL, and creatinine. Both urinary biomarkers were measured by using the high-performance liquid chromatography-tandem mass spectroscopy (HPLC MS/MS) technique. Urinary cotinine and NNAL were, respectively, measured by using Agilent 1100 Series with API 4000 and Agilent 1200 Series with Triple Quadrupole 5500, both manufactured by AB Sciex LLC (Framingham, MA, USA). The limits of detection for the urinary cotinine and NNAL levels of the mentioned methods are 0.27399 ng/mL and 0.1006 pg/mL, respectively. The rate-blanked and compensated Jaffe creatinine method was used to measure urinary creatinine by using the Automatic Analyzer 7600-210 made by Hitachi, Ltd. (Tokyo, Japan). All chemical measurements were conducted by the Korea Disease Control and Prevention Agency. Each of the urinary biomarker levels was expressed as a ratio of the urinary creatinine concentration.

### 2.2. Selection of Subjects

The subjects of this study are selected from the participants of the KNHANES VII based on their answers to the questionnaire and the result of their urinary biomarker analysis. Each participant has a unique individual identity code and a family identity code. The family identity code is the same for each member of a family.

To conduct this study, first, we excluded subjects with undetectable urinary cotinine and creatinine levels or those whose age and weight were not reported in the KNHANES VII database. Then, we limited the study subjects by considering non-smokers, including former smokers, based on their self-reporting declaration. Among these subjects, we selected those who are living with at least one active smoker in the same family. Thereafter, we grouped all the associated active smokers as the positive control, including 3203 subjects.

In this community, we excluded children aged below six because they were not eligible for urine biomarker analysis experiments; therefore, their records did not include the level of tobacco smoke exposure biomarkers. In the next step, to concentrate on selecting passive smokers with realistic tobacco smoke exposure by urinary level, we excluded the former smokers and adolescents who reported that they experienced direct smoking of cigarettes. Moreover, to control the outliers of the urinary cotinine levels, we tried to find a suitable cutoff value for urinary cotinine levels to distinguish smokers and non-smokers. According to Kim [23], the urine cotinine cutoff is 50–200 μg/L. Following the dataset of KNHANES VII, the average urinary creatinine level of the participants (20,756 subjects) during the period 2016–2018 was 1.52 g-creatinine/L. Therefore, the urine cotinine cutoff suggested by Kim [23] will be approximately 33–131 μg/g-creatinine.

**Figure 1:**
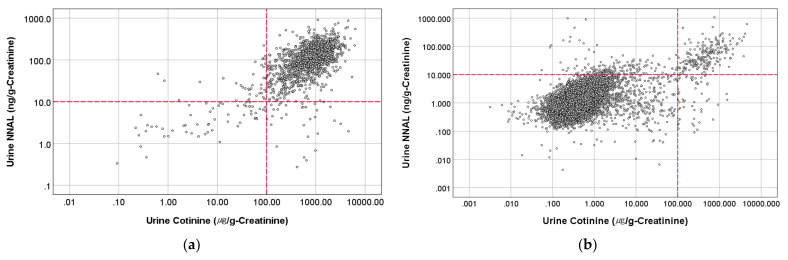
Condition of urinary NNAL and cotinine in (**a**) positive control (active smokers) and (**b**) non-smoker subjects.

On the other hand, we have plotted the urinary cotinine level versus urinary NNAL level for positive control and non-smokers, as presented in Figure 1. It became clear that around 90% of the positive control group (smokers) had cotinine and NNAL levels above 100 μg/g-creatinine and 10 ng/g-creatinine, respectively. The urine cotinine cutoff of 100 μg/g-creatinine is within the range suggested by Kim [23] and can be an acceptable cutoff value to distinguish smokers and non-smokers.

As presented in Figure 1, around 4% of the non-smokers’ urinary cotinine level exceeded the cut-off value of 100 μg/g-creatinine, while among them, 40% were self-reported to be former smokers. Therefore, the outlier may appear because of self-reporting error or including former smokers. Consequently, to remove these outliers in the last subject selection step, we have excluded subjects with a urine cotinine level above 100 μg/g-creatinine.

Table 1 summarizes the subject selection process. A total number of 2736 participants were subjected to passive ETS exposure. Among those who were subjected to passive ETS exposure, 870 subjects reported that they were exposed to the smoke of tobacco at home, at work, or in a public area, and were grouped as SHS exposure subjects. The rest of them were grouped as THS exposure subjects.

Table 2 presents the subjects’ socio-demographic information, along with their urinary cotinine level. Considering the population density in Korea, the selected subjects for both positive control and passive ETS exposure may have an acceptable regional coverage all over the country.

### 2.3. Analysis of Cigarette Smoke Composition and Inhalation Toxicity

From 2015 to 2016, the Ministry of Food and Drug Safety of the Republic of Korea gathered 400 packs of 5 cigarette products from 20 tobacco stores in 7 regions across the country at different time and season intervals, and investigated the amount of 45 components in the main smoke stream of each cigarette, including tobacco-specific nitrosamines (TSNAs), following the International Organization for Standardization (ISO) and Health Canada (HC) regime [12,24,25]. The selection of cigarettes proceeded following the ISO-8243 standard method [26]. The measurement methods are comprehensively described by the National Institute of Food and Drug Safety Evaluation [25].

Among the 45 components whose level in the cigarettes’ mainstream was investigated, we selected 26 ingredients, including nicotine, and other carcinogen and non-carcinogen compounds that were detectable and had sufficient accessible information on toxicity values for inhalation exposure from different sources of toxicity information. Table 3 summarizes the concentration of the selected 26 toxic compounds in cigarette smoke composition and their inhalation toxicity values, including the reference concentration (RfC) and inhalation unit risk (IUR).

### 2.4. Calculation of the Internal Estimated Daily Intake for Nicotine and Equivalent Smoked Cigarettes per Day

For each subject, the internal estimated daily intake (I-EDI) for nicotine (μg-nicotine/day) was approximated using Equation (1), where UCot is the level of urinary cotinine (μg-cotinine/g-creatinine), UCE is the daily creatinine excretion rate (g-creatinine/day), and F_UE_ is the ratio of urinary execration to total elimination. For nicotine, an F_UE_ of 0.11 was considered following Benowitz et al. [33]. MW_Nicotine_ and MW_Cotinine_ are the molecular weights of nicotine and cotinine, respectively.

(1) $$I-\mathrm{EDI}\;=\frac{\mathrm{UCot}\;\times \;\mathrm{UCE}}{F_{\mathrm{UE}}}\times \frac{{\mathrm{MW}}_{\mathrm{Nicotine}}}{{\mathrm{MW}}_{\mathrm{Cotinine}}}\;,$$

Following the results of Kang et al. [34] on Korean subjects, the UCE was estimated for each male and female subject through Equation (2). In Equation (2), A and BW are the subject’s age (year) and body weight (kg), respectively.

(2) $$\mathrm{UCE}\;=\left\{\begin{array}{c} \mathrm{For}\;\mathrm{men}:\;\frac{\left(23.4-0.09A\right)\times \;\mathrm{BW}}{1000}\\ \mathrm{For}\;\mathrm{women}:\;\frac{\left(18.0-0.08A\right)\times \;\mathrm{BW}}{1000} \end{array}\right.\;,$$

For each individual, the equivalent smoked cigarettes per day (CPD, Cig/day) was estimated by using Equation (3), where the Nicotine Avg. (μg/Cig) is the average of nicotine ingredients in the cigarette’s composition. As mentioned in Table 2, this parameter was considered as 470 μg/Cig.

(3) $$\mathrm{CPD}\;=\frac{I-\mathrm{EDI}}{\mathrm{Nicotine}\;\mathrm{Avg}.}\;,$$

### 2.5. Inhalation Exposure and Risk Assessment

Non-carcinogenic and carcinogenic inhalation exposure and risk were assessed following the US Environmental Protection Agency’s RAGS Supplemental Guidance for Inhalation Risk Assessment [35]. For each subject, the average daily dose (ADD, μg/m^3^) for non-carcinogenic inhalation exposure to each compound in the cigarette was estimated through Equation (4), where C_cig_ is the amount of the compound in the cigarette composition, Abs is the absorption rate of the compound, EF and ED are the exposure frequency (day/year) and duration (year), respectively, and IR is the inhalation ratio of the subject (m^3^/day). Considering the gender and age of the subject, IR is estimated by referring to the National Institute of Environmental Research [36,37]. AT is the averaging exposure time (days). For non-cancer inhalation exposure and risk assessment, AT is estimated as EF × ED. According to Marano et al. [20], Abs can have a range of 0.1 to 1.0. In this study, the maximum values of Abs and EF of 1.0 and 365 days, respectively, were used for exposure assessment.

(4) $$\mathrm{ADD}\;=\frac{C_{\mathrm{Cig}}\times \;\mathrm{CPD}\;\times \;\mathrm{Abs}\;\times \;\mathrm{EF}\;\times \;\mathrm{ED}}{\mathrm{IR}\;\times \;\mathrm{AT}}\;,$$

As shown in Equation (5), the hazard quotient (HQ) of each compound was calculated as the ratio of ADD to the reference concentration (RfC, μg/m^3^). For the whole cigarette composition, the hazard index (HI) was calculated by summing the HQs of the compounds.

(5) $$\mathrm{HI}\;=\sum_{i=1}^{n}{\mathrm{HQ}}_{i}=\sum_{i=1}^{n}\frac{\mathrm{ADD}}{{\mathrm{RfC}}_{i}}\;,$$

The accumulative lifetime average daily dose (LADD_Accumulative_, μg/m^3^) of carcinogenic inhalation exposure to each compound in the cigarette for each subject was calculated through Equation (6), where ADAF is the age-dependent adjustments factor. ADAFs of 10, 3, and 1 are assigned to subjects aged 0–2, 3–15, and above 16 years old, respectively. Since the IR changes significantly with age, ED and IR have been calculated for age groups of 0–2, 3–5, 6–8, 9–11, 12–15, and above 16 years old following the National Institute of Environmental Research [36,37]. For the carcinogenic inhalation exposure and risk assessment, ET and AT are considered to be 365 and 25,550 days, respectively.

(6) $${\mathrm{LADD}}_{\mathrm{Accumulative}}=\sum_{i=1}^{6}\;\frac{C_{\mathrm{Cig}}\times \;\mathrm{CPD}\;\times \;\mathrm{Abs}\;\times \;\mathrm{EF}}{\mathrm{AT}}\times {\left(\frac{\mathrm{ED}\;\times \;\mathrm{ADAF}}{\mathrm{IR}}\right)}_{i}\;,$$

Table 4 summarizes the values of ED, ADAF, and IR for each value of i in Equation (6).

The total excess lifetime cancer risk (Total ECR) for the cigarette composition is calculated by summing the excess lifetime cancer risk (ECR) of each compound calculated through Equation (7), where IUR_i_ is the inhalation unit risk of the compound i.

(7) $$\mathrm{Total}\;\mathrm{ECR}\;=\sum_{i=1}^{n}{\mathrm{ECR}}_{i}=\sum_{i=1}^{n}{\mathrm{LADD}}_{\mathrm{Accumulative}}\times {\;\mathrm{IUR}}_{i}\;,$$

### 2.6. Data Analysis

The normality of distributions of the urinary cotinine level and their natural logarithm value in both passive ETS exposure groups were tested by using Kolmogorov–Smirnov and Shapiro–Wilk with a significance level of α = 0.05. For all cases, the *p*-value was below 0.001, thereby showing that the urinary cotinine levels of the study groups were neither normal nor lognormal distributed. Accordingly, a nonparametric method of Mann–Whitney U test or Kruskal–Wallis H test was used, where applicable, to analyze the significance of differences in parameters. Spearman’s correlation coefficient (ρ) was calculated to analyze the correlation between the urinary cotinine levels in the passive ETS exposure groups with the ones related to the smokers who are living in the same family. All statistical analyses were performed with IBM^®^ SPSS^®^ Statistics version 25 (IBM Company, Armonk, NY, USA). The significance level was set at α = 0.05.

## 3. Results

### 3.1. Correlation between the Urinary Smoking Exposure Biomarker of Active and Passive Smokers Living in the Same Family

**Figure 2:**
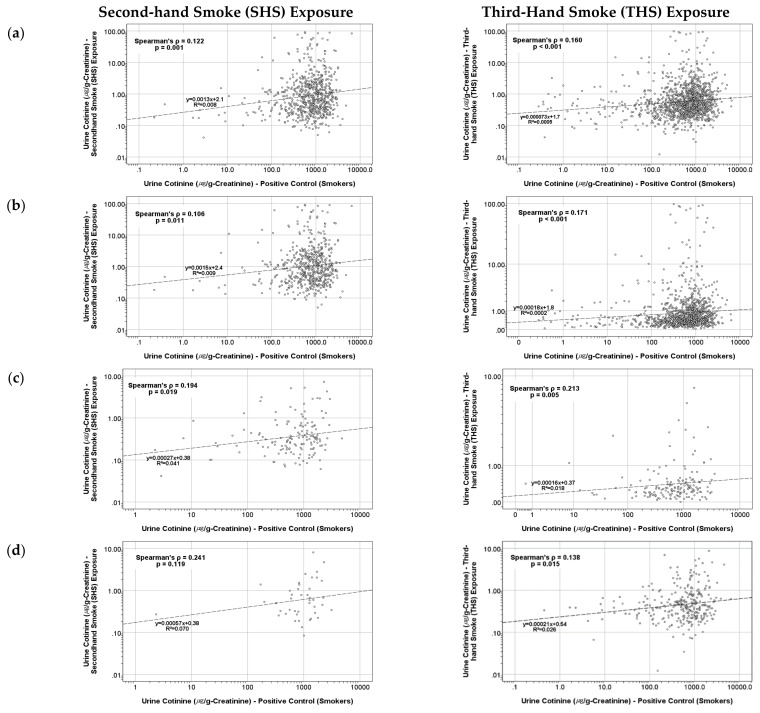
Correlation of urine cotinine levels of active and passive smokers living in the same family according to the age of the passive ETS exposure subjects: (**a**) all ages, (**b**) adults (age ≥19), (**c**) adolescents (ages 12–18), and (**d**) children (ages 6–11).

Figure 2 presents the correlation between the urinary cotinine levels of active smokers and passive ETS exposure subjects who are living in the same family. The analysis was performed for different age groups, i.e., children (ages 6–11), adolescents (ages 12–18), and adults (ages 19 and above), following the definitions and instructions provided by the KNHANES VII. In all cases, the urinary smoking exposure biomarker level in passive ETS exposure subjects had a positive linear association with the level in the smokers of the family. The association was significant for all age groups (*p* < 0.05) except for children among the SHS exposure group (*p* = 0.119). These results yield a statistically significant effect of the presence of a smoker in a family on the urinary smoking exposure biomarker of non-smokers.

### 3.2. Results of I-EDI for Nicotine and Equivalent Smoked Cigarettes per Day in Passive ETS Exposure Subjects

Table 5 summarizes the results of the calculated I-EDI for nicotine and equivalent smoked cigarettes per day in passive ETS exposure subjects considering a nicotine composition of 470 μg/Cig. The results yield that the urinary cotinine level in SHS exposures is equivalent to smoking an average of 1.2 cigarettes per month with an approximate nicotine composition of 0.5 mg/Cig, which is widely available in Korean markets. The equivalent smoking of cigarettes per month was higher in women among SHS exposure subjects to be about 1.5 cigarettes per month. These numbers are half for THS exposure subjects.

The maximum value for equivalent smoking cigarettes per month among SHS exposure subjects exceeds 10 cigarettes per month for subjects aged 6–10, which can be interpreted as half of the number of cigarettes in a standard-sized box. For SHS exposure subjects above 14 years old, the equivalent smoking cigarette per month exceeds 20 cigarettes per month, which equals one box of cigarettes per month.

Both nicotine I-EDI and equivalent CPD were significantly different for SHS and THS exposures (*p* < 0.001). Comparison of nicotine I-EDI and equivalent CPD among SHS exposure subjects was not significant between different genders (*p* = 0.878), while it was significant between different ages (*p* < 0.001). However, for THS exposure subjects, both nicotine I-EDI and equivalent CPD were significantly different when compared based on gender (*p* = 0.042) or age (*p* < 0.001).

### 3.3. Results of Hazard Quotient Assessment for the Composition of Cigarette Smoke

**Figure 3:**
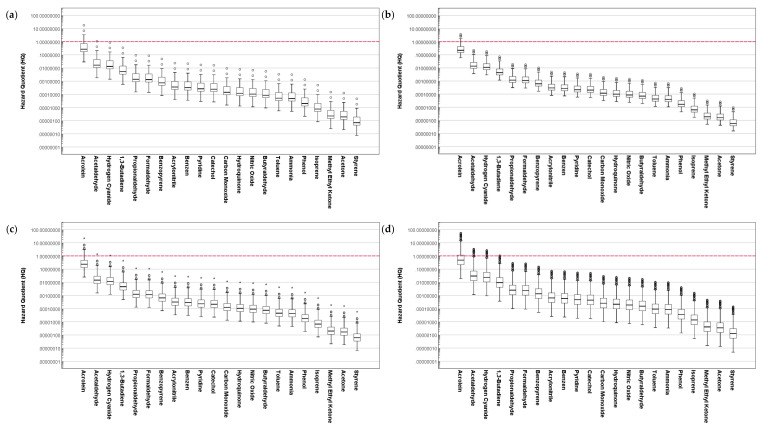
Assessment of hazard quotient for the composition of cigarette smoke to SHS exposure subjects based on age: (**a**) 6–10 years old, (**b**) 11–13 years old, (**c**) 14–18 years old, and (**d**) 19 years old and above.

**Figure 4:**
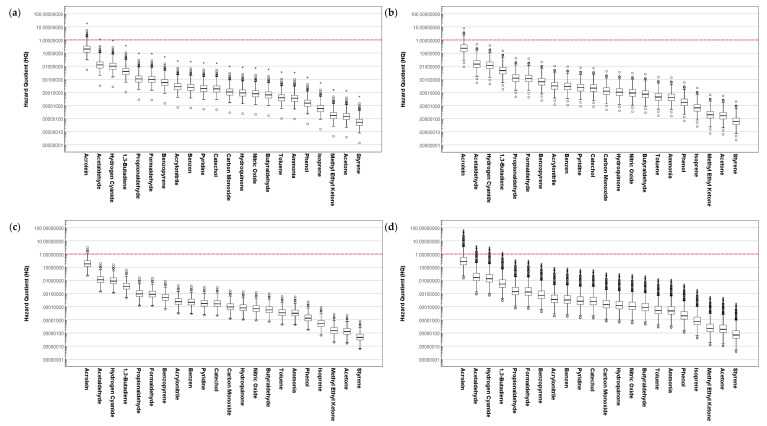
Assessment of hazard quotient for the composition of cigarette smoke to THS exposure subjects based on age: (**a**) 6–10 years old, (**b**) 11–13 years old, (**c**) 14–18 years old, and (**d**) 19 years old and above.

Figure 3 and Figure 4 demonstrate the assessed inhalation HQ of the composition of cigarette smoke for the different age groups of SHS and THS subjects. For each group, the distribution of HQ was significantly different when compared based on the hazardous substances in the cigarette smoke (*p* < 0.001).

For both SHS and THS subjects, the assessed HQ for the composition of cigarette smoke was significantly different when compared based on age (*p* < 0.001). When compared based on gender, the assessed HQ for the SHS subjects was not significantly different (*p* = 0.096), while it was statistically different for the THS subjects (*p* = 0.001).

For all age groups of SHS and THS, the distribution HQ of acrolein exceeded 1, which indicates a high likelihood of toxicological responses. Although acrolein has a relatively small concentration contribution in the composition of cigarette smoke, it also has a small RfC, thus leading to a high HQ assessment. Along with acrolein, the distribution of HQ assessed for acetaldehyde and hydrogen cyanide in cigarette smoke for all age groups of SHS and THS subjects exceeded 0.1, which suggests the requirement of more efficient management in controlling passive ETS exposures.

### 3.4. Results of Excess Lifetime Cancer Risk Assessment for the Composition of Cigarette Smoke

**Figure 5:**
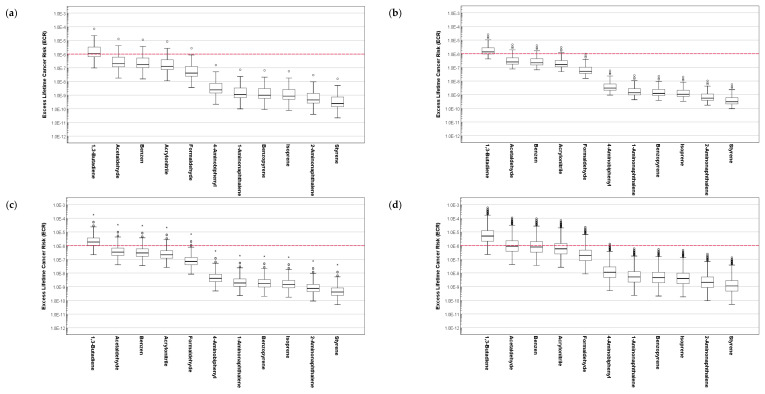
Assessment of excess lifetime cancer risk for the composition of cigarette smoke to SHS exposure subjects based on age: (**a**) 6–10 years old, (**b**) 11–13 years old, (**c**) 14–18 years old, and (**d**) 19 years old and above.

**Figure 6:**
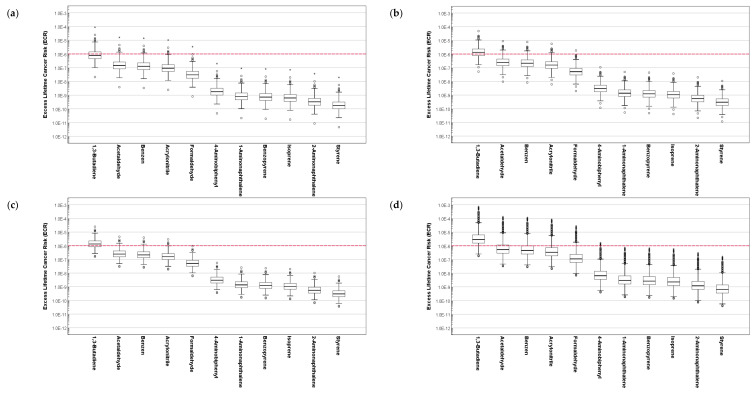
Assessment of excess lifetime cancer risk for the composition of cigarette smoke to THS exposure subjects based on age: (**a**) 6–10 years old, (**b**) 11–13 years old, (**c**) 14–18 years old, and (**d**) 19 years old and above.

Figure 5 and Figure 6 present the assessed inhalation ECR of the composition of cigarette smoke for different age groups of SHS and THS subjects. Similar to the assessed HQ, the distribution of ECR for each group was significantly different when compared based on the hazardous substances in the cigarette smoke (*p* < 0.001). For both SHS and THS subjects, the assessed ECR for the composition of cigarette smoke was significantly different when compared based on age and gender (*p* < 0.001).

For all age groups of SHS and THS subjects, the average of the assessed ECR for 1,3-butadiene exceeded 1 × 10^−6^, which indicates a potential incidence of cancer from inhalation exposure route to be above one person among one million people.

Regarding SHS subjects, the average likelihood of cancer incidence was higher in women (ECR_1,3-butadiene_ = 1.88 × 10^−5^) compared to men (ECR_1,3-butadiene_ = 9.35 × 10^−6^). The ECR of 1,3-butadiene for SHS subjects aged 6–10 was estimated to be 3.99×10^−6^. Similar results were obtained for the THS subjects. ECRs of 1,3-butadiene for male, female, and subjects aged 6–10 in the THS exposure group were 4.59 × 10^−6^, 8.89 × 10^−6^, and 1.65 × 10^−6^, respectively. Along with 1,3-butadiene, the distribution of ECR assessed for acetaldehyde, benzene, and acrylonitrile in cigarette smoke for all age groups of SHS and THS subjects exceeded 1 × 10^−6^, which yields a high potential likelihood for cancer incidences in passive ETS exposures. These results suggest the requirement of the provision of protection against passive ETS exposures to lower the likelihood of cancer incidences, especially for children and adolescents.

### 3.5. Results of Hazard Index and Total Excess Lifetime Cancer Risk for ETS Exposure Subjects

**Figure 7:**
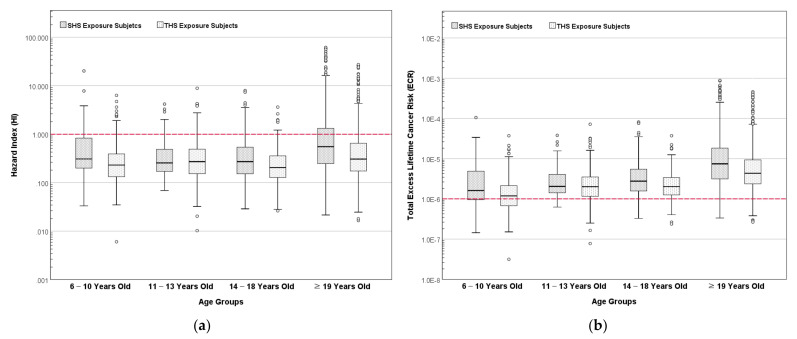
Assessment of (**a**) hazard index and (**b**) total excess lifetime cancer risk for the composition of cigarette smoke to ETS exposure subjects based on age.

Figure 7 presents the results of assessed HI and total ECR for ETS exposure subjects. The results of HI and total ECR distribution were significantly different for the SHS and THS subjects (*p* < 0.001). For both SHS and THS subjects, the distributions of HI and total ECR were significantly different when compared based on age (*p* < 0.001).

Among the SHS subjects, a comparison of the distribution of HI between men and women showed no statistically significant difference (*p* = 0.372), while the difference of total ECR based on gender among the SHS subjects was significantly different (*p* < 0.001). Similar results were obtained for the THS subjects. The comparison of the distribution of HI between men and women among the THS subjects showed no statistically significant difference (*p* = 0.076), while the distribution of the total ECR was significantly different when compared based on the subjects’ gender (*p* < 0.001).

The results indicate a relatively high HI and total ECR for all age groups among the SHS and THS subjects. Among the SHS subjects, the average HI for women was 2.07, which was higher than that of men who were assessed to have an average HI of 1.32. The mean of assessed HI for the SHS subjects aged 6–11 was 1.14.

The total ECR assessed for women among SHS subjects was 2.83 × 10^−5^, which was higher than that of men at 1.40 × 10^−5^. The average of the assessed total ECR for SHS subjects aged 6–11 was 6.00 × 10^−6^. A similar trend of results was observed for the THS subjects, which indicates that the average HI and total ECR for passive ETS exposure exceeded 1 and 1 × 10^−6^, respectively. This suggests a high likelihood of both adverse non-carcinogenic and carcinogenic health effects due to the passive ETS exposure, especially in women and children who are living with smokers.

## 4. Discussion

Our study assessed passive ETS exposure and its risk of carcinogenic and non-carcinogenic health effects through a nationwide survey. The study results yield a significant positive correlation between the urinary cotinine level, a biomarker of smoking exposure, in smokers and non-smokers in a family. These results are consistent with the results of Jeong et al. [38]. Using the subjects’ urinary cotinine level, we have calculated the I-EDI of nicotine, followed by estimating the equivalent CPD. This method enabled us to also assess the carcinogenic and non-carcinogenic risks of both SHS and THS exposure. Hence, our study may be able to contribute toward developing study protocols aiming to investigate the contribution of THS to overall tobacco smoke exposure, as suggested by Mahabee-Gittens et al. [39].

The study results also showed a relatively high HI (>1) and total ECR (>1 × 10^−6^) in women and children among both SHS and THS exposure, which are consistent with several other studies that have also investigated the effects of passive ETS exposure on children. Passive smoking exposure can cause a wide range of non-carcinogen symptoms, including coughing, runny nose, and increase in heart rate in children who are living with smoking parents [40]. Nadhiroh et al. [41] showed that parental SHS exposure can also be associated with a lower head circumference. Nevertheless, while usually parents make efforts to limit their children’s exposure to ETS, the health effect of both SHS and THS exposure is unavoidable [42]. Therefore, programs for encouraging smokers to proceed with smoking cessation are essential in order to protect women and children from passive ETS exposure [12,17].

Our study should be considered in light of several limitations. The database of KNHANES VII includes the results of a nationwide investigation. However, it does not include repetitive results of an individual, which means that the results are based on a one-time investigation. It is also not possible to distinguish the exact number of people who are causing passive ETS exposure in a family. According to Vitória [43], SHS in a family can be caused by the smoking habits of parents, other family members, and guests, while KNHANES VII may limitedly include the results of the whole family. It is also not possible to estimate the exact EF or distinguish the location of exposure.

Moreover, results of cigarette smoke composition provided by the National Institute of Food and Drug Safety Evaluation may not include all possible existing carcinogen and non-carcinogen substances in cigarette smokers. The investigation and analysis of cigarette smoke composition are being repeated periodically in order to provide more accurate results. Meanwhile, the composition of cigarette smoke may differ even from one cigarette to another in the same brand following the harvesting and processing of the tobacco leaves [12].

KNHANES VII includes the results of urinary cotinine and NNAL as biomarkers for exposure to tobacco smoke. Considering the results of cigarette smoke composition, which include the measured level of nicotine, the only biomarker that we could use for conducting our study was urinary cotinine. Hence, the inclusion of other appropriate urinary or blood biomarkers, such as trans-3’-hydroxycotinine, in KNHANES can be useful for conducting more accurate daily intake estimation in future studies [19].

Accordingly, more in-depth studies with enhanced controlling methods are required in order to provide a better understanding of the carcinogenic and non-carcinogenic health effects of SHS and THS exposure. In particular, similar nationwide scaled studies can be conducted periodically and results can be used for detailed assessment of risk associated with ETS.

## 5. Conclusions

In this study, we assessed the urinary cotinine levels of SHS and THS exposure subjects and their correlation with the biomarker level in the smokers living with them through the nationwide survey of KNHANES VII in the Republic of Korea. It became clear that there is a statistically meaningful positive association between them. We also assessed the I-EDI of the SHS and THS subjects and evaluated the equivalent CPD to assess the carcinogenic and non-carcinogenic risks of SHS and THS exposure. The results indicated a relatively high likelihood for both carcinogenic and non-carcinogenic health effect incidences, especially in women and children. In the composition of the cigarette smoke, 1,3-butadiene and acrolein substances had the highest contribution to the HI and total ECR. Accordingly, to protect non-smokers against passive ETS exposure, it is suggested to make national policies and regulations for reducing the concentrations of such toxic chemicals from the cigarette smoke composition as a short-term action. Meanwhile, designing, offering, and managing efficient smoking cessation programs for smokers, focusing on those who are living with children and adolescents in the same family, can also be suggested as long-term actions.

## Figures and Tables

**Table 1:** Subject selection process from the KNHANES VII dataset.

Selection Step	Subjects Description	Number of Subjects	Level of Urinary Cotinine (μg/g-Creatinine) Mean ± SD (Min–Max)
0	Everyone with valid data in the KNHANES VII dataset	17,686	189.7 ± 503.3 (0.0014~6446)
1	All non-smokers (including former smokers) in the KNHANES VII dataset	14,333	27.5 ± 182.2 (0.0014~5324)
2	Non-smokers and former smokers who are living in a family, including at least one smoker	3233	35.4 ± 222.2 (0.012~4571)
3	Excluding subjects with urinary cotinine levels above 100 μg/g-creatinine	2736	1.86 ± 7.19 (0.012~93.8)

**Table 2:** Socio-demographic information and urinary cotinine level of the subjects.

Items (Unit)		Positive Control (Active Smokers)		Passive ETS Exposure			
		*n*	Value (% or Mean ± SD)	Second-Hand Smoke (SHS)Exposure		Third-Hand Smoke (THS)Exposure	
				*n*	Value (% or Mean ± SD)	*n*	Value (% or Mean ± SD)
Gender	Male	2704	84.4%	210	24.1%	409	21.9%
	Female	499	15.6%	660	75.9%	1457	78.1%
Age Range (Years)	6–10	-	-	53	6.1%	348	18.6%
	11–13	-	-	66	7.6%	136	7.3%
	14–18	-	-	117	13.4%	115	6.2%
	≥19	3203	100%	634	72.9%	1267	67.9%
Body Weight (kg)		3201	69.8 ± 13.1	870	58.8 ± 14.5	1866	53.5 ± 15.3
Body Mass Index (kg/m^2^)		3198	24.2 ± 3.68	870	23.0 ± 4.25	1864	22.1 ± 4.42
Residence Region	Seoul	554	17.3%	116	13.3%	354	19.0%
	Busan	207	6.5%	67	7.7%	106	5.7%
	Daegu	148	4.6%	44	5.1%	78	4.2%
	Incheon	184	5.7%	55	6.3%	122	6.5%
	Gwangju	95	3.0%	27	3.1%	55	2.9%
	Daejeon	109	3.4%	33	3.8%	67	3.6%
	Ulsan	73	2.3%	33	3.8%	31	1.7%
	Sejong	53	1.7%	21	2.4%	32	1.7%
	Gyeonggi-do	814	25.4%	188	21.6%	498	26.7%
	Gangwon-do	104	3.2%	24	2.8%	53	2.8%
	Chungcheongbuk-do	96	3.0%	24	2.8%	46	2.5%
	Chungcheongnam-do	122	3.5%	34	3.9%	55	2.9%
	Jeollabuk-do	99	3.1%	20	2.3%	60	3.2%
	Jeollanam-do	106	3.3%	32	3.7%	79	4.2%
	Gyeongsangbuk-do	190	5.9%	69	7.9%	89	4.8%
	Gyeongsangnam-do	177	5.5%	60	6.9%	91	4.9%
	Jeju Special Self-governing Province	72	2.2%	23	2.6%	50	2.7%
Education Level	Elementary School or Lower	373	11.6%	264	30.3%	744	39.9%
	Middle School	311	9.7%	148	17.0%	177	9.5%
	High School	1243	38.8%	25	2.9%	446	23.9%
	College or Higher	1127	35.2%	178	20.5%	459	24.6%
	No Answer (Unknown)	149	4.7%	255	29.3%	40	2.1%
Urinary Cotinine (μg/g-creatinine)		3203	912.7 ± 772.2	870	2.82 ± 8.90	1866	1.42 ± 6.20

**Table 3:** The concentration of the selected toxic compounds in cigarette smoke composition and their inhalation toxicity values.

Compound	Concentration in Main Stream of Cigarette Smoke (μg/Cig)		Inhalation Toxicity Value		
	Mean ± SD	Range (Min–Max)	Reference Concentration (mg/m^3^)	Inhalation Unit Risk (per μg/m^3^)	Reference
Nicotine	470 ± 44.7	400–500	-	-	-
1-Aminonaphthalene	0.0068 ± 0.002	0.0056–0.0095	-	5.14 × 10^−4^	[20]
1,3-Butadiene	20.1 ± 4.75	15.0–26.1	0.002	0.00017	[27]
2-Aminonaphthalene	0.0027 ± 0.001	0.0020–0.0043	-	5.14 × 10^−4^	[20]
4-Aminobiphenyl	0.0013 ± 0.0002	0.0011–0.0016	-	0.006	[27]
Acetaldehyde	285.0 ± 44.1	224.7–327.2	0.009	2.20 × 10^−6^	[28]
Acetone	113.5 ± 11.0	104.5–127.4	30.9	-	[29]
Acrolein	10.3 ± 1.24	8.80–11.4	2.00 × 10^−5^	-	[28]
Acrylonitrile	1.38 ± 0.70	0.80–2.40	0.002	2.90 × 10^−4^	[27]
Ammonia	6.38 ± 1.0	5.30–7.80	0.07	-	[29]
Benzene	18.7 ± 4.31	13.0–23.8	0.03	2.90 × 10^−5^	[27]
Benzopyrene	0.003 ± 0.001	0.0017–0.0045	2.00 × 10^−6^	0.0011	[28]
Butyraldehyde	16.3 ± 2.81	13.8–19.5	0.1	-	[30]
Carbon Monoxide	6280 ± 1535.3	3700–7500	23	-	[27]
Catechol	65.8 ± 12.5	47.0–80.5	0.14	-	[31]
Formaldehyde	10.0 ± 2.62	8.20–14.3	0.004	1.30 × 10^−5^	[28]
Hydrogen Cyanide	20.3 ± 3.16	15.9–23.8	0.0008	-	[28]
Hydroquinone	20.2 ± 2.96	15.5–23.5	0.088	-	[31]
Isoprene	122.6 ± 26.0	91.7–158.3	8.4	2.20 × 10^−8^	[30]
Methyl Ethyl Ketone	21.6 ± 1.91	19.6–24.1	5	-	[28]
Nitric Oxide	93.7 ± 27.6	52.3–129.9	0.47	-	[27]
Phenol	7.32 ± 2.64	3.10–9.70	0.19	-	[32]
Propionaldehyde	21.5 ± 3.83	17.4–25.7	0.008	-	[28]
Pyridine	1.76 ± 0.47	1.00–2.20	0.0035	-	[32]
Styrene	1.34 ± 0.40	0.80–1.80	1	5.70 × 10^−7^	[28,32]
Toluene	29.4 ± 4.94	22.4–35.9	0.3	-	[27]

**Table 4:** The concentration of the selected toxic compounds in cigarette smoke composition and their inhalation toxicity values.

*i*-Value	Age Range	ED (Years)	ADAF	IR (m^3^/day)		Reference
				Male	Female	
1	0–2	2	10	9.80	9.25	[36,37]
2	3–5	3	3	10.96	9.62	
3	6–8	3	3	11.39	10.08	
4	9–11	3	3	12.49	11.65	
5	12–15	4	3	15.55	12.66	
6	≥16	Current Age–16	1	16.43	13.64	

**Table 5:** Results of nicotine I-EDI and equivalent CPD for ETS exposure subjects.

Variable	Subjects	Groups						
		All	Gender		Age Range (Years)			
			Male	Female	6–10	11–13	14–18	≥19
Nicotine I-EDI (μg-nicotine/day) Mean ± SD (min–max)	SHS Exposure	20.3 ± 61.6 (0.27–643.7)	15.8 ± 37.1 (0.84–335.2)	21.8 ± 67.5 (0.27–643.7)	10.4 ± 27.1 (0.27–185.1)	5.65 ± 8.44 (0.84–51.0)	10.8 ± 34.0 (0.36–335.2)	24.4 ± 69.7 (0.30–643.7)
	THS Exposure	9.77 ± 44.0 (0.06–718.7)	7.41 ± 35.9 (0.06–678.4)	10.4 ± 46.0 (0.11–718.7)	4.1 ± 11.2 (0.06–187.3)	5.94 ± 11.3 (0.11–100.4)	4.01 ± 6.04 (0.28–48.6)	12.3 ± 52.7 (0.17–718.7)
Equivalent CPD (Cig/day) Mean ± SD (min–max)	SHS Exposure	0.04 ± 0.13 (0.0006–1.37)	0.03 ± 0.08 (0.002–0.71)	0.05 ± 0.14 (0.0006–1.37)	0.02 ± 0.06 (0.0006–0.40)	0.01 ± 0.02 (0.002–0.11)	0.02 ± 0.07 (0.001–0.71)	0.05 ± 0.15 (0.0007–1.37)
	THS Exposure	0.02 ± 0.09 (0.0001–1.53)	0.02 ± 0.08 (0.0001–1.44)	0.02 ± 0.10 (0.0002–1.53)	0.01 ± 0.02 (0.0001–0.40)	0.01 ± 0.02 (0.0002–0.21)	0.01 ± 0.01 (0.0006–0.10)	0.03 ± 0.11 (0.0004–1.53)

## Data Availability

Publicly available datasets were analyzed in this study. These data can be found here: https://knhanes.kdca.go.kr/knhanes/main.do (accessed on 22 October 2021).

## Abbreviations

**Abbreviation**	**Meaning**
Abs	Absorption Rate
ADAF	Age-Dependent Adjustments Factor
ADD	Average Daily Dose
AT	Averaging Exposure Time
CPD	Cigarette per Day
ECR	Excess Lifetime Cancer Risk
ED	Exposure Duration
EF	Exposure Frequency
ETS	Exposure to Environmental Tobacco Smoke
FCTC	Framework Convention on Tobacco Control
HC	Health Canada
HI	Hazard Index
HPLC MS/MS	High-Performance Liquid Chromatography-Tandem Mass Spectroscopy
HQ	Hazard Quotient
I-EDI	Internal Estimated Daily Intake
IR	Inhalation Ratio
ISO	International Organization for Standardization
IUR	Inhalation Unit Risk
KNHANES	Korea National Health and Nutrition Examination Survey
LADD	Lifetime Average Daily Dose
NNAL	4-(Methylnitrosamino)-1-(3-Pyridyl)-1-Butanol
RfC	Reference Concentration
SHS	Second-hand Smoke
THS	Third-hand Smoke
TSNAs	Tobacco-specific Nitrosamines
WHO	World Health Organization
